# Ghrelin Promotes Proliferation and Inhibits Differentiation of 3T3-L1 and Human Primary Preadipocytes

**DOI:** 10.3389/fphys.2019.01296

**Published:** 2019-10-11

**Authors:** Hui Miao, Hui Pan, Linjie Wang, Hongbo Yang, Huijuan Zhu, Fengying Gong

**Affiliations:** Key Laboratory of Endocrinology of National Health Commission, Department of Endocrinology, Peking Union Medical College Hospital, Chinese Academy of Medical Sciences and Peking Union Medical College, Beijing, China

**Keywords:** ghrelin, proliferation, differentiation, 3T3-L1 preadipocytes, human primary preadipocytes

## Abstract

**Objective:**

Ghrelin is a 28-amino-acid peptide that regulates energy hemostasis, glucose and lipid metabolism. We aimed to explore the effects of ghrelin on the proliferation and differentiation of 3T3-L1 and human primary preadipocytes.

**Methods:**

3-(4,5-Dimethylthiazol-2-yl) 2,5-diphenyl tetrazolium bromide (MTT) spectrophotometry, Oil Red O staining, intracellular glycerol-3-phosphate dehydrogenase (G-3-PDH) assays and semiquantitative reverse transcription polymerase chain reaction were used to investigate the action of ghrelin.

**Results:**

Ghrelin (0.01–1000 ng/ml) significantly increased the numbers of 3T3-L1 cells, and the maximum stimulatory effect was observed with the 10 ng/ml ghrelin treatment for 24 h (*p* < 0.05). Ghrelin also promoted the proliferation of human primary preadipocytes from 24 h (*p* < 0.05) to 48 h (*p* < 0.05) at a concentration of 1000 ng/ml. Further investigation showed that IGF-1 levels were notably increased in ghrelin-treated 3T3-L1 and human preadipocytes, and IGF-1 antibody was capable to attenuate this stimulatory action of ghrelin (all *p* < 0.05). Additionally, ghrelin significantly suppressed the differentiation of 3T3-L1 and human primary preadipocytes; 10 ng/ml ghrelin notably downregulated G-3-PDH activities in 3T3-L1 cells on day 3 and in human cells from days 4 to 12 following differentiation (all *p* < 0.05), and the intracellular lipoprotein lipase mRNA levels were lower than that of the controls (*p* < 0.05). Further investigation showed that the mRNA levels of peroxisome proliferator-activated receptor γ2 (PPARγ2) and CCAAT/enhancer binding protein α (C/EBPs) were also suppressed in ghrelin-treated human differentiating adipocytes.

**Conclusion:**

Ghrelin promotes the proliferation of 3T3-L1 and human primary preadipocytes by increasing the expression of IGF-1. Ghrelin inhibits murine and human adipocyte differentiation by downregulating PPARγ2 and C/EBPα levels, consequently leading to decreased lipid accumulation and lipogenic enzymes expression.

## Introduction

Obesity and its related disorders, such as type 2 diabetes, dyslipidemia, and cardiovascular disease has increased in prevalence in recent years and has become a vital health concern worldwide (Corica et al., 2015; Martin et al., 2015). Obesity is mainly due to excessive energy intake and less expenditure, thereby leading to accumulation of fat (Wright and Aronne, 2012), but the mechanism of obesity has not been fully elucidated. It has been reported that increased adipocyte number (hyperplastic growth) and adipocyte volume (hypertrophic growth) are of great importance in the occurrence and development of obesity (Berry et al., 2014).

Preadipocytes are recruited from multipotent stem cells and regularly undergo mitotic clonal expansion, thereby upregulating adipocyte number. When treated with appropriate cytokines or hormones, preadipocytes are growth-arrested and induced to differentiate (Young et al., 1995; Lane and Tang, 2005). In the process of differentiation, some transcription factors have been reported to activate adipogenesis. Two known factors are peroxisome proliferator-activated receptor γ2 (PPARγ2) and CCAAT/enhancer binding protein α (C/EBPs), which cooperate as a complex to promote preadipocyte differentiation and are required for the maintenance of adipocytes (Rosen et al., 2002; Imai et al., 2004). Moreover, these transcription factors are associated with activating the expression of adipocyte-specific enzymes, which are associated with lipogenesis and lipid retention. For instance, lipoprotein lipase (LPL) reveals lipid storage in the early stages of differentiation, whereas fatty acid synthase (FAS), acetyl-CoA carboxylase (ACC), and glycerol-3-phosphate dehydrogenase (G-3-PDH) are involved in triglyceride metabolism in late stages (Ailhaud, 1996; Lenhard, 2011; Mracek et al., 2013; Kersten, 2014).

Factors influencing adipogenesis and lipid retention have been receiving substantial attention from researchers, and several hormones, such as insulin, glucocorticoid and thyroxine, have been found to affect proliferation and differentiation of preadipocytes (Geloen et al., 1989; Obregon, 2008). Ghrelin, a 28-amino-acid peptide mainly secreted by the gastric fundus mucosa, was first described in 1999 and is the endogenous ligand of the growth hormone secretagogue receptor 1a (GHS-R1a) (Kojima et al., 1999). Ghrelin stimulates appetite and food intake by activating Neuropeptide Y (NPY) neurons and agouti-related peptides (AGRP) in the hypothalamus (Warchol et al., 2014), and a negative association of ghrelin and body mass index (BMI) has been found in both rats and human (Tschop et al., 2001; Williams et al., 2006). Recent studies have also found that ghrelin functions directly in adipocytes. Ghrelin stimulates adipogenesis of mature rat adipocytes from perigonadal, epididymal and parametrial regions, mainly by increasing adipogenic factors and lipogenic enzymes (Choi et al., 2003; Porteiro et al., 2013). It also suppresses lipolysis in rat visceral adipocytes through the activation of phosphoinositide 3-kinase (PI3K) (Baragli et al., 2011).

Several studies have shown that ghrelin stimulates the adipogenesis of 3T3-L1 preadipocytes (Choi et al., 2003; Churm et al., 2017), but the detailed mechanism of ghrelin has not been fully clarified. Insulin-like growth factor-1 (IGF-1) is a protein secreted by the liver after stimulation from growth hormone (GH), and the GH/IGF-1 axis mediates mitogenic and functional activities in cells throughout the body (Ashpole et al., 2015). The IGF-1 signal activates the phosphatidylinositol 3-kinase (PI3K)/Akt and extracellular regulated protein kinases (ERK)/mitogen-activated protein kinase (MAPK) pathways, thereby regulating cellular proliferation and differentiation (Kasprzak and Adamek, 2012; Gao et al., 2013; Ye et al., 2018). Ghrelin has been shown to promote the growth and differentiation of adipocytes via the MAPK and PI3K/Akt pathways (Kim et al., 2004; Liu et al., 2015), but whether this effect is achieved through insulin-like growth factor 1 (IGF-1) is not clear. Additionally, most studies have focused on mouse cells, but according to our knowledge, few studies have illustrated the regulatory effect of ghrelin on human preadipocytes. Therefore, the purpose of our study was to determine the effect of ghrelin on the proliferation and differentiation of mouse 3T3-L1 and human primary preadipocytes.

## Materials and Methods

### Materials

3T3-L1 preadipocytes were received as a kind gift from the Department of Genetics, Institute of Life Sciences, Peking University, Beijing. Human primary preadipocytes were obtained from the abdominal subcutaneous adipose tissues (SATs) of three patients undergoing surgery (appendicitis, cholelithiasis, and colon benign neoplasm). The clinical details of these three patients are listed in Supplementary Table S1. The study was approved by the ethics committee of Peking Union Medical College Hospital (No. JS-1093). All patients provided informed consent before adipose tissue was obtained during the surgery. Rat ghrelin was purchased from Sigma, United States. Human IGF-1 and IGF-1 antibodies were kind gifts from WHO and NIDDK, NIH, United States, respectively. Trizol RNA kit was purchased from Promega, United States. Ultraviolet-visible spectrophotometer was purchased from Hitachi, Japan. Applied Biosystems PCR System was purchased from Promega, United States.

### Cell Culture and Differentiation of 3T3-L1 and Human Primary Preadipocytes

Human subcutaneous adipose tissues were prepared in 5–10 g samples, then rinsed by 1 × phosphate buffered saline (PBS) three times. The remaining connective tissue and blood vessels were cleared. The adipose tissues were digested with 5 ml collagenase (2 mg/ml) for 40 min at 37°C. Samples were filtered through a 25 μm mesh and centrifuged at 600 *g* for 5 min. The supernatant was discarded, and the cell pellet was re-suspended in DMEM/F12 medium. Unfiltered tissues were digested once more according to the steps above. Cells were collected and counted before being seeded in DMEM/F12 (HyClone, United States) with the addition of 10% FBS (Yuan Heng Sheng Ma Biotechnology Research Institute, China), then cultured in a 5% CO_2_ 95% air atmosphere incubator. The inverted phase contrast microscope (Olympus, Japan) was used to observe the growth and differentiation state. The cells were induced to differentiate in AIM-V (Gibco, United States) medium supplemented with 0.5 μM insulin, 0.25 μM dexamethasone, 0.2 nM thyroxine, and 0.5 nM 3-isobutyl-1-methylxanthine (IBMX) (Sigma, United States). The differentiation medium was refreshed every 2 days.

#### Proliferation of 3T3-L1 and Human Primary Preadipocytes

Preadipocytes were seeded in a 96-well plate with a density of 5 × 10^3^/well in 10% FBS/DMEM medium for 12 h, then transferred to AIM-V medium. After 4 days, 3T3-L1 preadipocytes were added with 0.01, 0.1, 1, 10, 100, 1000 ng/ml ghrelin, and human cells were added with 0.1, 10, 1000 ng/ml ghrelin. After 4, 8, 24, 48 and 72 h, the preadipocytes were rinsed twice using 1 × phosphate buffered saline (PBS). 3-(4,5-dimethylthiazol-2-yl) 2,5-diphenyl tetrazolium bromide (MTT, Sigma, United States) solution was added to each well with a final concentration of 1 mg/mL, and the cells were incubated for 4 h. Then, 100 μL dimethylsulfoxide solution was added, and the cells were shaken for 10 min. Afterward, optical density (OD) value was recorded using an ELISA reader (Anthos Labtec, Austria) at a wavelength of 492 nm. IGF-1 antibody was added after treatment with ghrelin (10 and 1000 ng/ml in rat and human cells, respectively). Experiments were repeated six times with individual octuplicate samples in each experiment.

3T3-L1 preadipocytes were seeded in a 96-well plate with a density of 8 × 10^3^/well in 10% FBS/DMEM medium for 12 h. The cells were rinsed twice using 1 × phosphate buffered saline (PBS), then transferred to X-Vivo 15 medium (Lonza, United States) medium for 4 days. After treatment with 1, 10, and 100 ng/ml ghrelin for 24 and 48 h, 10 μL of the Cell Counting Kit-8 (CCK-8, Sagecreation, Beijing) solution was added to each well of the plate. The cells were then incubated for 2 h in the incubator, and the absorbance was measured at 450 and 620 nm using a microplate reader (Thermo, United States).

3T3-L1 and human preadipocytes were seeded at a density of 5 × 10^3^/well as mentioned above. After 4 days, 3T3-L1 preadipocytes were added with 0.25, 1, 2.5, 5, 10, 25, and 100 ng/ml IGF-1, and human primary preadipocytes were added with 1, 10, 100 ng/ml IGF-1 for. After 24 h, MTT assays were conducted to determine the effect of IGF-1 on proliferation.

#### Differentiation of 3T3-L1 and Human Primary Preadipocytes

3T3-L1 and human preadipocytes were seeded at a density of 5 × 10^3^/well in 96-well plate and the differentiation was performed based on the protocol mentioned above. After 3 days in AIM-V medium, cells were treated with 0.1, 10, 1000 ng/ml ghrelin and 1, 10, 100 ng/ml IGF-1 in AIM-V differential medium for 4, 8, 12 days for human primary preadipocytes and 1, 3, 6 days for 3T3-L1 cells due to a faster differentiation rate in murine preadipocytes. Oil Red O (Ameresco, United States) staining was conducted as described previously (Zhu et al., 2006). In brief, preadipocytes were washed twice using 1× PBS, following treatment with 10% fresh formaldehyde for 1 h at room temperature for fixation, and then, the cells were stained with 0.6% (w/v) filtered Oil red O solution for 2 h and photographed using an inverted phase contrast microscope. 100 μL isopropanol was added to dissolve oil red O dyes, and the OD value was measured using an ELISA reader at a wavelength of 492 nm. Experiments were repeated six times with octuplicate samples in each experiment.

Enzyme G-3-PDH is specifically expressed in differentiating adipocytes, so the activity of Glycerol-3-phosphate dehydrogenase (G-3-PDH) was measured to observe the triglyceride synthesis action of cells (Rauchová et al., 2004). Murine and human cells were treated with 10 ng/ml ghrelin and IGF-1 for the indicated time, then washed twice using 1 × PBS, and 0.5 ml of lysis buffer (50 mM Tris, 1 mM EDTA, 0.1 mM β-mercaptoethanol) was added to each well. Subsequently, the cells were scraped and sonicated in ice for 30 s to generate cell lysis. 300 μL samples was transferred to 1.5 ml assay buffer (100 mM Triethanolamine hydrochloride, 2.5 mM EDTA, 0.12 mM NADH, 0.1 mM β-mercaptoethanol) to detect G-3-PDH activity. G-3-PDH activity was tested using an ultraviolet spectrophotometer at a wavelength of 340 nm. 100 μL dihydroxyacetone phosphate buffer was added and activity was remeasured at 340 nm. 1 unit of G-3-PDH activity represents the oxidation amount of 1.0 nM NADH per minute. Experiments were replicated three times with triplicate samples in each experiment.

#### Semiquantitative Reverse Transcription-PCR (RT-PCR) Analysis

The differentiating cells were treated with 10 ng/ml ghrelin and IGF-1 for 8 days. Total RNA was extracted using a Trizol RNA kit (Promega, United States), and 2 μg RNA was reverse transcribed with an RT-PCR system including M-Mul reverse transcriptase (Promega, United States), RNasin (Promega, United States), Oligo (dT) primer (Promega, United States) and deoxyribonucleoside Triphosphate (TaKaRa, Japan). 2 μl RT products (cDNA) were amplified with human LPL, PPARγ2, C/EBPα, β-actin primers and mouse LPL, IGF-1 primers as shown in Table 1. 10 μl RT-PCR products were electrophoresed in 2% agarose gel in Tris-acetate-EDTA buffer. The gel was then stained with ethidium bromide and photographed using an Alphalmager M2200 (AlphaInnotech, United States). Expression changes were determined by the density ratio of the target genes to mGAPDH or β-actin in 3T3-L1 and human primary preadipocytes, respectively. Experiments were replicated three times with triplicate samples in each experiment.

**TABLE 1:** Primers used in RT-PCR.

**Genes**	**Primers**	**Length (bp)**
hLPL	5′-ACA CAG CTG AGG ACA CTT GC-3′ 5′-CAC TGG GTA ATG CTC CTG AG-3′	227
hPPAR γ2	5′-GAT ACA CTG TCT GCA AAC ATA TCA CAA-3′ 5′′-CCA CGG AGC TGA TCC CAA-3′	91
hC/EBPα	5′-GCA AGG CCA AGA AGT CGG TGG AC-3′ 5′-TGC CCA TGG CCT TGA CCA AGG AG-3′	252
β-Actin (human)	5′-GTG GGG CGC CCC AGG CAC CA–3′ 5′-CTT CCT TAA TGT CAC GCA CGA TTT C-3′	540
mLPL	5′-ATG GAG AGC AAA GCC CTG C-3′ 5′-AGT CCT CTC TCT GCA ATC AC-3′	770
mIGF-1	5′-ATG GGG AAA ATC AGC AGT C-3′ 5′-AGG TCT TGT TTC CTG CAC-3′	413
mGAPDH	5′-TCC ACT CAC GGC AAA TTC AAC G-3′ 5′-TAG ACT CCA CGA CAT ACT CAG C-3′	145

#### Quantitative Real-Time RT-PCR (qRT-PCR) Analysis

3T3-L1 preadipocytes were cultured in X-Vivo 15 medium and MesenCult^TM^-ACF Plus Medium (STEMCELL, Canada) based on the protocol mentioned above. Concentrations of 1, 10, and 100 ng/ml ghrelin (abcam, United Kingdom, ab120231) were administrated for 24 h to preadipocytes and 8 days to differentiating cells. RT-qPCR analysis was performed using SYBR Premix Ex Taq (TaKaRa, Japan) and an ABI7500 PCR system (Applied Biosystems, San Francisco, CA, United States) as previously described (Yan et al., 2017). The total RNA was extracted using an EZNA total RNA kit (Omega Biotek, United States, Lot R6834-01). 1 μg RNA was reverse transcribed to cDNA using the PrimeScript^TM^ RT reagent Kit (TaKaRa, Japan). RT-qPCR was conducted in 3T3-L1 preadipocytes and adipocytes to assess the expression of proliferation and differentiation-related genes. The primer sequences of target genes are listed in Table 2. PPIA was used as the internal control, and the relative expression of each target gene was calculated using the formula 2^–ΔΔ*Ct*^. RT-qPCR was duplicated in 2 wells in a final volume of 20 μL.

**TABLE 2:** Primers used in qRT-PCR.

**Genes**	**Primers**	**Length (bp**
mPPIA	5′-GCTGGACCAAACACAAACGG-3′ 5′-TCCTGGACCCAAAACGCTC-3′	139
mcyclin D1	5′-GCGTACCCTGACACCAATCTC-3′ 5′-CTCCTCTTCGCACTTCTGCTC-3′	183
mcyclin E1	5′-GTGGCTCCGACCTTTCAGTC-3′ 5′-CACAGTCTTGTCAATCTTGGCA-3′	101
mcyclin B2	5′-GCCAAGAGCCATGTGACTATC-3′ 5′-CAGAGCTGGTACTTTGGTGTTC-3′	114
mBAX	5′-TGAAGACAGGGGCCTTTTTG-3′ 5′-AATTCGCCGGAGACACTCG-3′	140
mBcl-2	5′-GCAGAGATGTCCAGTCAGC-3′ 5′-CCCACCGAACTCAAAGAAGG-3′	129
mBcl-xL	5′-GGTATTGGTGAGTCGGATTGC-3′ 5′-CGGCTCTCGGCTGCTG-3′	132
mPPAR γ2	5′-GCAGGAGCAGAGCAAAGAG-3′ 5′-GGACACCATACTTGAGCAGAG-3′	158
mC/EBPα	5′-CAAGAACAGCAACGAGTACCG-3′ 5′-GTCACTGGTCAACTCCAGCAC-3′	124
mLPL	5′-GCCTGAGTTGTAGAAAGAATCG-3′ 5′-GTTGTGTTGCTTGCCATCC-3′	173
mACC	5′-CAGCATCTCTAACTTCCTTCAC-3′ 5′-CGAGCCATTCATTATCACTACG-3′	163

### Statistical Analysis

All statistical analyses were performed using SPSS 10.0 for Windows, and all values were expressed as the mean ± standard deviation (SD). A *t*-test was used to analyze for two groups, and one-way ANOVA was used for three or more groups. The Mann–Whitney *U* or Kruskal–Wallis test was used if data were still not normally distributed. *p* < 0.05 was considered significant for all analyses.

## Results

### Morphological Features of 3T3-L1 Preadipocytes and Human Primary Preadipocytes

**FIGURE 1:**
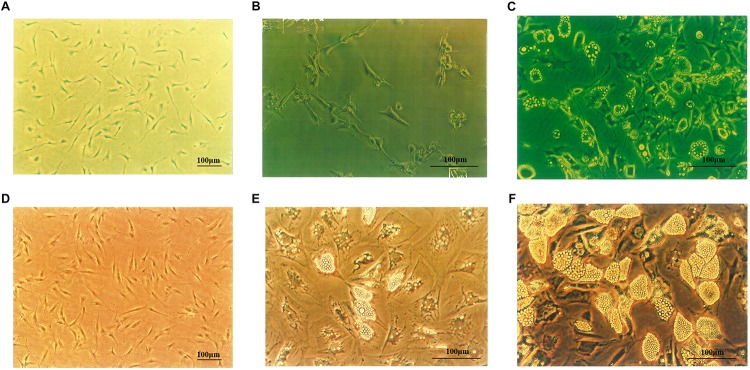
3T3-L1 and human primary preadipocytes proliferation and differentiation. **(A)** 3T3-L1 preadipocyte on Day 4 (×100). Adipocytes on Day 4 **(B)** and Day 14 **(C)** after inducing differentiation (×200). **(D)** Human primary preadipocyte on Day 8 (×100). Adipocytes on Day 10 **(E)** and Day 18 **(F)** after inducing differentiation (×200). Magnification: ×100 or ×200.

3T3-L1 cells (Figure 1A) were fusiform or had multiple protrusions, with round nucleus in the center of the cytoplasm. On day 4 following differentiation (Figure 1B), small lipid droplets could be observed in the cytoplasm. With longer differentiation periods, the number of the lipid droplets increased, small lipid droplets merged into large lipid droplets and the cells became oval or circular. On the 14^th^ day of differentiation, large lipid droplets could be notably observed, and 95 to 98% of the cells differentiated to mature adipocytes (Figure 1C). As shown in Figure 1D, the morphology of human primary preadipocytes was similar to murine cells. On the 10^th^ day of differentiation, lipid droplets became larger and pushed the nucleus to one side of the cell (Figure 1E). As depicted in Figure 1F, more than 80% of the human cells accumulated lipid droplets in the cytoplasm.

### Ghrelin and IGF-1 Stimulated the Proliferation of Mouse 3T3-L1 Preadipocytes and Human Primary Preadipocytes

**FIGURE 2:**
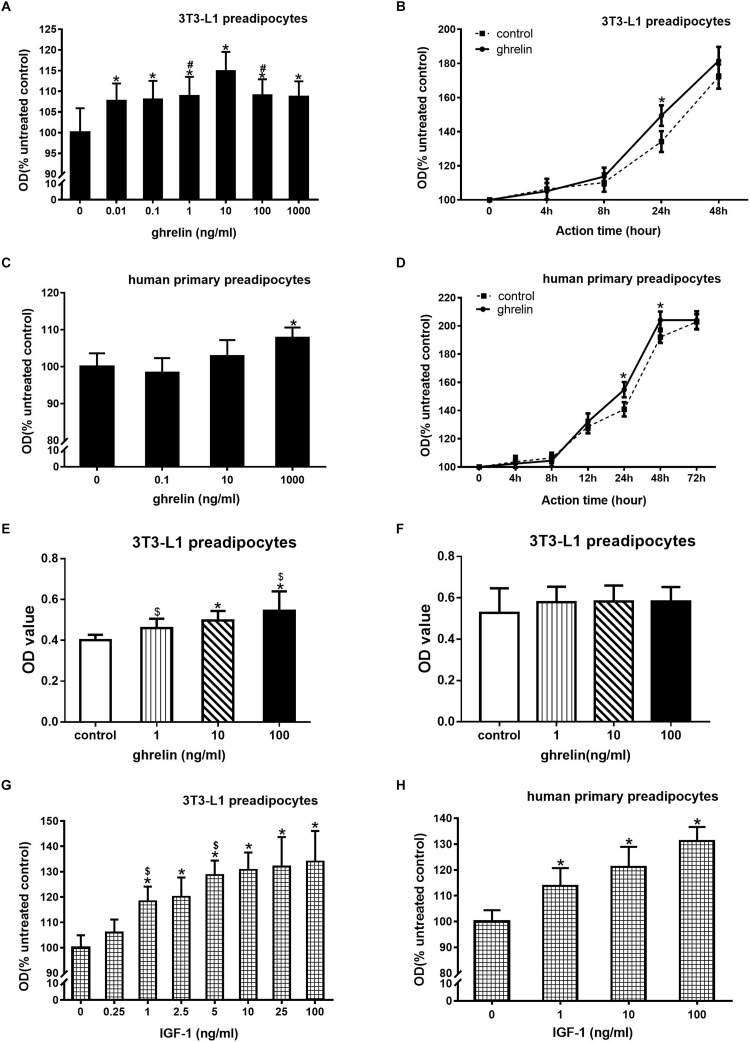
Ghrelin and IGF-1 stimulated the proliferation of mouse 3T3-L1 preadipocytes and human primary preadipocytes. Effect of ghrelin on 3T3-L1 preadipocytes at indicated concentrations **(A)** and time **(B)**. Effect of ghrelin on human primary preadipocytes at indicated concentrations **(C)** and time **(D)**. CCK-8 measurement of 3T3-L1 preadipocytes for 24 h **(E)** or 48 h **(F)**. Effect of ghrelin on 3T3-L1 preadipocytes at indicated concentrations **(G)** and time **(H)**. The data are presented as the mean ± SD for six **(A,C,G,H)** or three **(B,D,E,F)** independent experiments with eight individual wells in each experiment. ^∗^*p* < 0.05 vs. control group, #*p* < 0.05 vs. 10 ng/ml ghrelin group, $*p* < 0.05 in two groups.

3T3-L1 preadipocytes were treated with 0.01–1000 ng/ml ghrelin for 24 h. As presented in Figure 2A, 0.01 ng/ml ghrelin significantly promoted cell growth compared with the control group (107.7 ± 4.2 vs. 100.0 ± 5.9, *p* < 0.05), and this effect was further enhanced with the increase in ghrelin concentrations. The maximum promotion effect was observed at 10 ng/ml, which was up to 114.9% of the control group (*p* < 0.05). When the concentration of ghrelin was increased to 100 and 1000 ng/ml, its promotional effect was slightly weakened but was still higher than that of the control groups (*p* < 0.05).

To investigate the time curve of ghrelin on 3T3-L1 preadipocyte proliferation, 3T3-L1 preadipocytes were treated with 10 ng/ml ghrelin for 4–48 h. As shown in Figure 2B, the OD value of the control groups gradually increased during the 4–48 h period, which indicated the appropriate growth of 3T3-L1 cells. After treating these cells with ghrelin, the OD values of the cells also gradually increased and were consistently higher than that of the controls from 4 to 48 h, but the significant stimulatory effect was only observed at 24 h (149.4 ± 4.6 vs. 134.2 ± 6.1%, *p* < 0.05).

We observed that ghrelin promoted the proliferation of 3T3-L1 preadipocytes, and a similar result was also noted in human primary preadipocytes. Human primary preadipocytes were treated with 0.1–1000 ng/ml ghrelin for 24 h. As shown in Figure 2C, only 1000 ng/ml ghrelin slightly but significantly enhanced the proliferation of human primary preadipocytes (107.8 ± 2.8 vs. 100.0 ± 3.6, *p* < 0.05). This stimulatory effect was not observed at lower concentrations (0.1 and 10 ng/ml). Figure 2D showed the time curve of ghrelin on human primary preadipocytes proliferation, and the OD value of human cell controls also gradually upregulated which was consistent with the observations of mouse 3T3-L1 cells. After adding 1000 ng/ml ghrelin to these cells, the OD value of the cells further increased from 4 to 72 h, and they became significantly higher than that of the control group from 24 h (154.8 ± 5.4 vs. 140.9 ± 5.1, *p* < 0.05) to 48 h (204.1 ± 6.1 vs. 192.1 ± 4.2, *p* < 0.05), but this stimulatory effect disappeared at 72 h.

CCK-8 was used to further evaluate the effect of ghrelin on the proliferation of 3T3-L1 preadipocytes. As shown in Figures 2E,F, 1, 10, and 100 ng/ml ghrelin stimulated cell proliferation at 24 h (Figure 2E, *p* < 0.05), but the concentrations showed no significant difference at 48 h compared with the control group (Figure 2F). These results were consistent with the MTT assay as shown in Figures 2A,B.

3T3-L1 preadipocytes were treated with 0.25–100 ng/ml ghrelin for 24 h (Figure 2G). Similar to the administration of ghrelin, 0.25 ng/ml IGF-1 could significantly enhance cell growth compared with the control group (105.9 ± 5.2 vs. 100.0 ± 4.9, *p* < 0.05), and this effect was concentration-dependent. The maximum promotion effect was observed at 100 ng/ml, which was up to 134% compared with the control group (*p* < 0.05). IGF-1 also presented a similar concentration-dependent stimulatory effect in human cells (Figure 2H), with the maximum effect of 131.2% at 100 ng/ml.

### Ghrelin Upregulated the Expression of IGF-1 in 3T3-L1 and Human Preadipocytes

**FIGURE 3:**
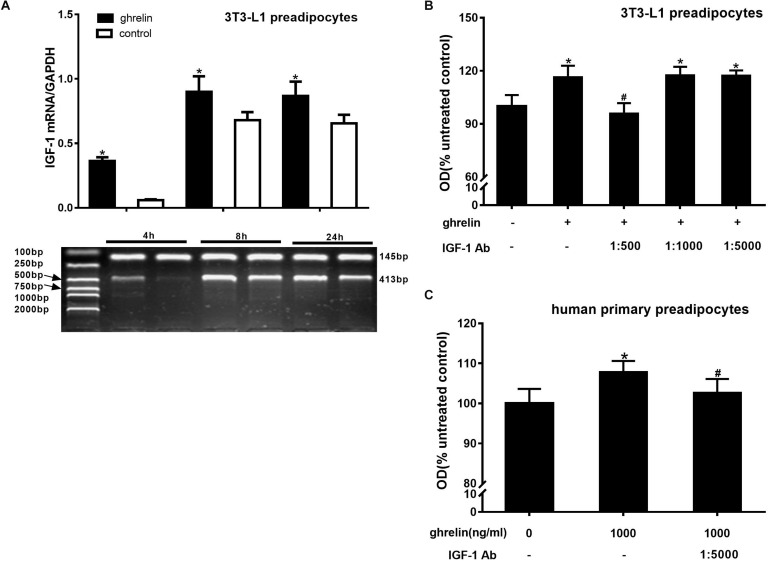
Ghrelin upregulated the expression of IGF-1 in 3T3-L1 and human preadipocytes. Effects of ghrelin on IGF-1 mRNA levels in 3T3-L1 preadipocytes **(A)**. Effect of IGF-1 antibody on the proliferation of 3T3-L1 preadipocytes **(B)** and human primary preadipocytes **(C)**. The data are presented as the mean ± SD for three independent experiments with eight individual wells in each experiment. ^∗^*p* < 0.05 vs. 0 ng/ml ghrelin group, #*p* < 0.05 vs. ghrelin group.

IGF-1 is a growth factor known to promote cell proliferation. To determine whether IGF-1 is involved in the proliferation of 3T3-L1 preadipocytes, the cells were treated with 10 ng/ml ghrelin for 4, 8, and 24 h, and the levels of IGF-1 mRNA were assessed using RT-PCR. As presented in Figure 3A, ghrelin significantly increased the expression of IGF-1 mRNA at 4, 8, and 24 h, which was 6.15 times that of the of controls at 4 h (*p* < 0.05), and 1.32-fold of that at both 8 h (*p* < 0.05) and 24 h (*p* < 0.05), suggesting that IGF-1 may be involved in the stimulatory proliferation of ghrelin in 3T3-L1 cells. To further confirm the hypothesis, the various dilutions of IGF-1 antibodies (1:500, 1:1000, 1:5000) were added together with 10 ng/ml ghrelin. As presented in Figure 3B, the stimulatory effect of ghrelin was attenuated only with the 1:500 IGF-1 antibody dilution by 17.8% (95.7 ± 6.0 vs. 116.4 ± 6.5; *p* < 0.05), whereas no significant changes were observed at the lower dilutions of 1:1000 (*p* > 0.05) and 1:5000.

Consistent with the observation in 3T3-L1 preadipocytes, the OD value of human primary preadipocytes also decreased after mixing with 1000 ng/ml ghrelin and 1:5000 IGF-1 antibody, indicating that the promotional effect of ghrelin on human preadipocytes was also through IGF-1. However, compared with the mouse cells, a low concentration (1:5000) of IGF-1 antibody was capable to attenuate the stimulatory action of 1000 ng/ml ghrelin (102.6 ± 3.5 vs. 107.8 ± 2.8; *p* = 0.0175) in human cells (Figure 3C). These results suggest that ghrelin affects the proliferation of mouse and human preadipocytes directly by upregulating IGF-1 expression.

**FIGURE 4:**
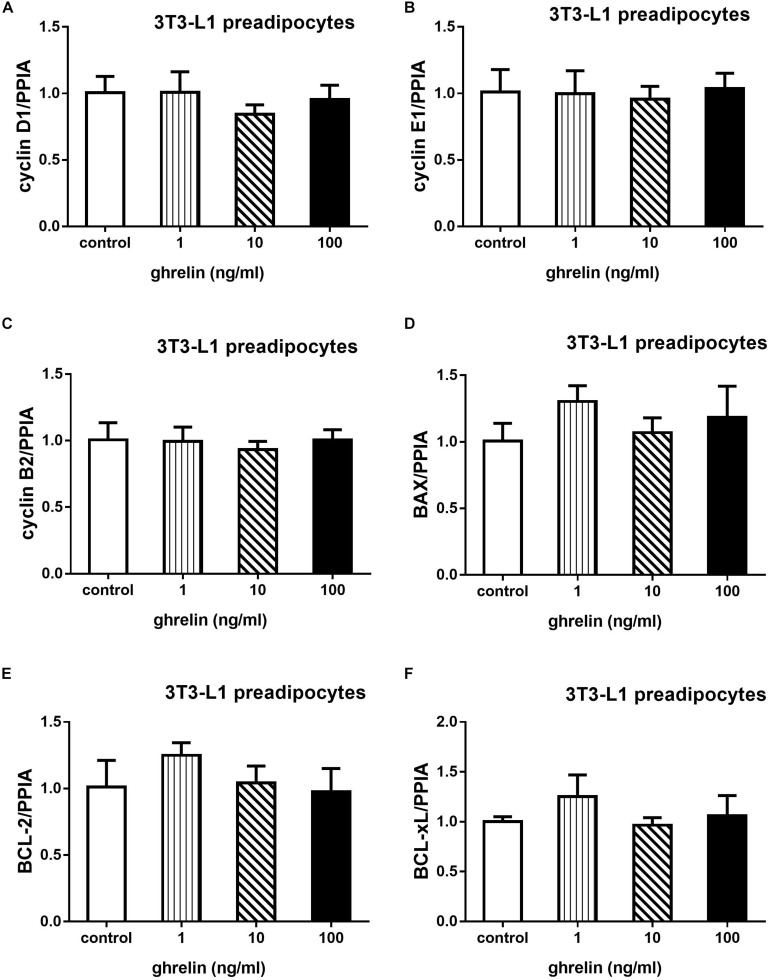
Effects of ghrelin on proliferation and apoptosis-related genes expression in 3T3-L1 preadipocytes. The mRNA levels of cyclin D1, E1, B2, and apoptosis genes BAX, Bcl-2, Bcl-xL were determined by RT-qPCR analysis **(A–F)**. The data are presented as the mean ± SD for three independent experiments with three wells in each experiment. ^∗^*p* < 0.05 vs. 0 ng/ml ghrelin group.

3T3-L1 preadipocytes were treated with 1, 10, and 100 ng/ml for 24 h, then qRT-PCR was conducted to explore whether ghrelin can affect cell proliferation and apoptosis. As shown in Figure 4, cell proliferation-related genes including cyclin D1, E1, B2 and apoptosis-related genes including BAX, Bcl-2, Bcl-xL were measured. Compared with the control groups, the expression of cyclins and apoptosis genes showed no significant difference after administrating ghrelin. The results suggest that ghrelin-induced 3T3-L1 cell proliferation may not be mediated through the genes we investigated in the present study.

### Ghrelin Suppressed While IGF-1 Stimulated the Differentiation of 3T3-L1 and Human Adipocytes

**FIGURE 5:**
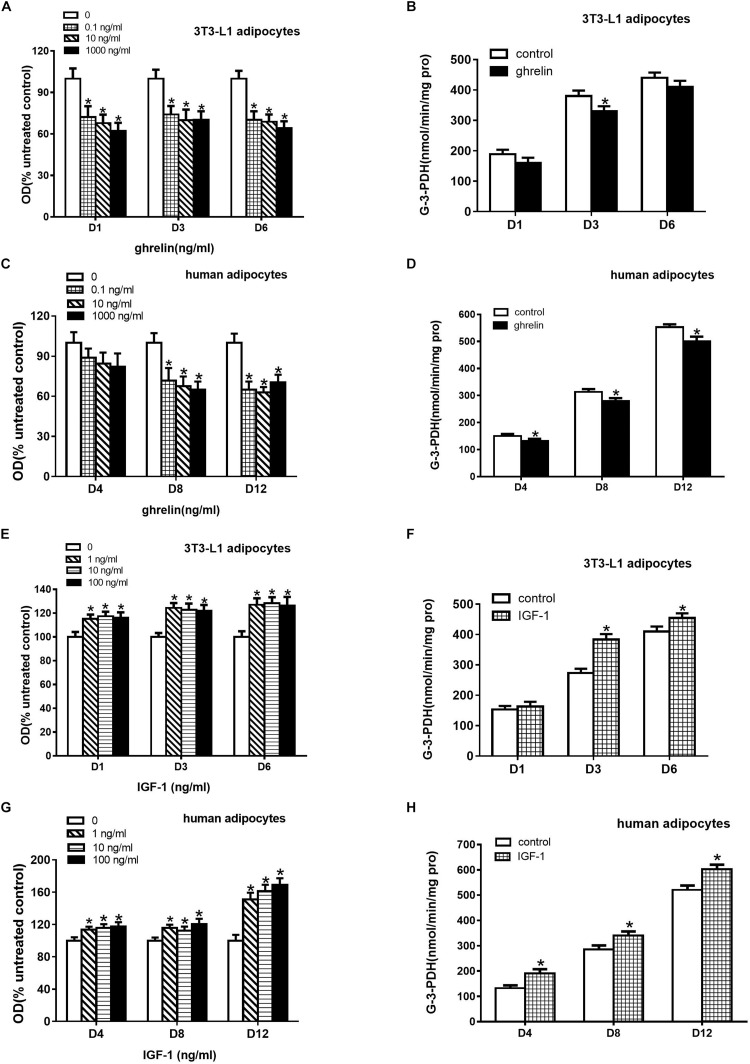
Ghrelin suppressed while IGF-1 stimulated the differentiation of 3T3-L1 and human primary preadipocytes. Differentiation was measured based on Oil Red O staining revealing triglyceride **(A,C,E,G)** and G-3-PDH activity **(B,D,F,H)**. Preadipocytes were treated with ghrelin **(A–D)** and IGF-1 **(E–H)**. The data are presented as the mean ± SD for three independent experiments with eight **(A,C,E,G)** or three **(B,D,F,H)** individual wells in each experiment. ^∗^*p* < 0.05 vs. control group.

Oil Red O staining can indirectly assess triglycerides in the cytoplasm of differentiated adipocytes. To investigate the effect of ghrelin on the differentiation of 3T3-L1 preadipocytes, these cells were treated with 0.1, 10, and 1000 ng/ml ghrelin for 1, 3, and 6 days. In this study as depicted in Figure 5A, we found that 0.1 ng/ml ghrelin significantly suppressed the OD values on the 1^st^ day after differentiation by 27.7% compared with the control group (*p* < 0.05). The inhibitory effect was observed at 10 and 1000 ng/ml ghrelin on days 1, 3 and 6 as well, but this effect was not further enhanced with the increase of doses and the extension of action time.

As the enzyme G-3-PDH is specifically expressed in differentiating adipocytes, we further investigated the effect of ghrelin on G-3-PDH activity. 10 ng/ml ghrelin was added to 3T3-L1 preadipocytes in a serum-free differential medium for 1, 3 and 6 days, and G-3-PDH activity was measured. As shown in Figure 5B, ghrelin could suppress G-3-PDH enzyme activities as expected, but it was only significantly decreased by 13.2% on the 3^rd^ day after differentiation (*p* < 0.05).

The experiments discussed above suggested that ghrelin inhibited the differentiation of 3T3-L1 preadipocytes based on triglyceride content and G-3-PDH activity. Consistent with the results of the mouse 3T3-L1 cells, the OD values of the human differentiating adipocytes also decreased with administration of 0.1 to 1000 ng/ml ghrelin for 4, 8, and 12 days (Figure 5C), but the significant results were observed only on days 8 and 12 following the differentiation (*p* < 0.05).

Figure 5D showed the activity of G-3-PDH in human differentiating adipocytes. After treating with 10 ng/ml ghrelin in a differential medium, the G-3-PDH activity of the cells reduced by 12.4% (150.4 ± 7.2 vs. 131.7 ± 8.1, *p* < 0.05), 10.5% (313.2 ± 10.7 vs. 280.4 ± 10.1, *p* < 0.05) and 9.3% (552.9 ± 10.4 vs. 501.4 ± 16.4, *p* < 0.05) on days 4, 8, and 12, respectively.

3T3-L1 preadipocytes were treated with 1, 10, 100 ng/ml IGF-1 for 1, 3, and 6 days. In contrast to the observation of ghrelin, as shown in Figure 5, IGF-1 enhanced the OD values (Figure 5E) and G-3-PDH activity (Figure 5F) of 3T3-L1 cells on days 1, 3, and 6. The stimulatory effect was also observed in human adipocytes, as depicted in Figures 5G,H. These results indicated that IGF-1 promoted the differentiation of both murine and human adipocytes.

### Ghrelin Inhibited the LPL mRNA Levels in 3T3-L1 and Human Differentiating Adipocytes

**FIGURE 6:**
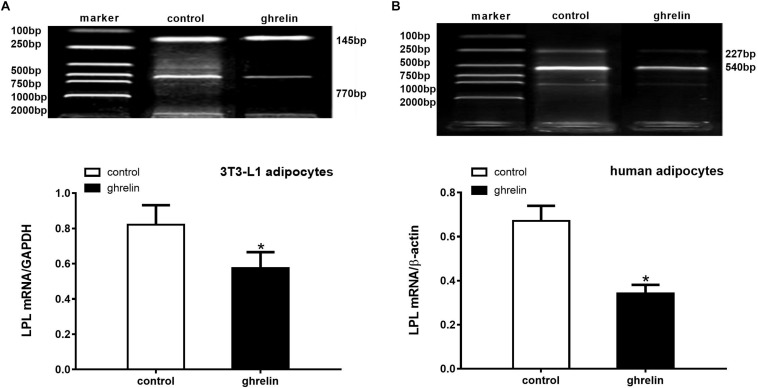
Ghrelin inhibited the LPL mRNA levels in 3T3-L1 and human differentiating adipocytes. Levels of LPL mRNA in 3T3-L1 **(A)** and human adipocytes **(B)** were measured by RT-PCR after treatment with 10 ng/ml ghrelin. LPL (770 bp) and GAPDH (145 bp) in 3T3-L1 preadipocytes, as well as LPL (227 bp) and β-actin (540 bp) in human adipocytes are shown with molecular markers in the upper panel of **(A,B)**. In the lower panel, data were calculated using the density ratio of LPL to GAPDH **(A)** and β-actin PCR products **(B)**. The data are presented as the mean ± SD for three independent experiments. ^∗^*p* < 0.05 vs. control group.

Because LPL is an essential enzyme expressed in the early stages of adipocyte differentiation, it has been widely used to investigate the effect of hormones on the differentiation of preadipocytes. To determine whether ghrelin can modulate the expression of this enzyme in mouse and human adipocytes, LPL mRNA levels were measured after adding ghrelin for 8 days following differentiation. As presented in Figure 6, we found that LPL mRNA decreased by 30.2% (0.574 ± 0.092 vs. 0.822 ± 0.110, *p* < 0.05) in 3T3-L1 adipocytes (Figure 6A) and by 49.0% (0.342 ± 0.039 vs. 0.671 ± 0.069, *p* < 0.05) in human adipocytes (Figure 6B) compared with the control groups, which indicated that ghrelin significantly inhibited the lipid metabolism of mouse and human differentiating adipocytes.

### Ghrelin Suppressed the Expression of PPARγ2 and C/EBPα mRNA in Human Differentiating Adipocytes

**FIGURE 7:**
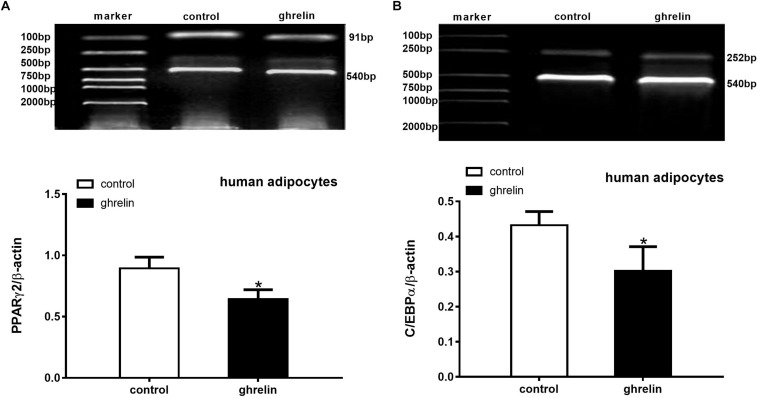
Ghrelin suppressed the expression of PPARγ2 and C/EBPα mRNA in human differentiating adipocytes. Levels of PPARγ2 **(A)** and C/EBPα **(B)** mRNA in human differentiating adipocytes were measured using RT-PCR after treatment with 10 ng/ml ghrelin. The data are presented as the mean ± SD for three independent experiments. ^∗^*p* < 0.05 vs. control group.

PPARγ2 and C/EBPα are two important transcription factors that can activate and mediate synergistically in the differentiation of preadipocytes. To clarify the effect of ghrelin on these adipogenic markers, the expression of PPARγ2 (Figure 7A) and C/EBPα (Figure 7B) were analyzed on day 8 after inducing differentiation. In accordance with the expectations, the levels of PPARγ2 and C/EBPα mRNA were markedly suppressed by 28.2% (0.642 ± 0.077 vs. 0.894 ± 0.091, *p* < 0.05) and 30.1% (0.302 ± 0.069 vs. 0.432 ± 0.039, *p* < 0.05) compared with the controls, suggesting that PPARγ2 and C/EBPα were involved in the inhibitory differentiation of ghrelin in human differentiating adipocytes.

### Ghrelin Downregulated the Expression of PPARγ2 and ACC mRNA in 3T3-L1 Adipocytes

**FIGURE 8:**
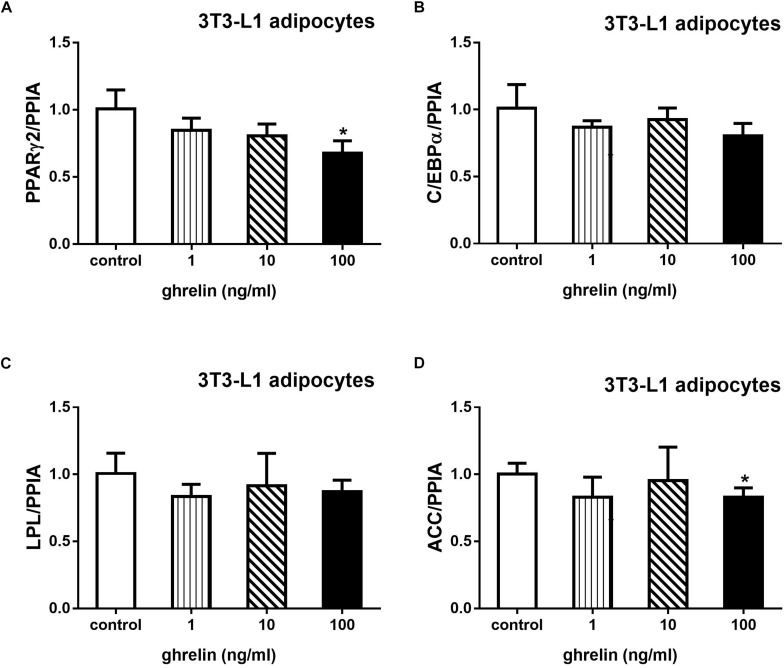
Ghrelin downregulated the expression of PPARγ2 and ACC mRNA in 3T3-L1 differentiating adipocytes. The mRNA levels of PPARγ2 **(A)**, C/EBPα **(B)**, LPL **(C)**, and ACC **(D)** were determined by determined by RT-qPCR analysis. The data are presented as the mean ± SD for three independent experiments with three wells in each experiment. ^∗^*p* < 0.05 vs. 0 ng/ml ghrelin group.

Acetyl-CoA carboxylase is an essential enzyme for fatty acid synthesis. 3T3-L1 preadipocytes were treated with 1, 10, and 100 ng/ml ghrelin for 8 days after inducing to differentiation. As depicted in Figure 8, 100 ng/ml ghrelin suppressed mRNA levels of PPARγ2 (Figure 8A) and ACC (Figure 8D) compared with the control groups (*p* < 0.05).

## Discussion

Ghrelin is a 28-amino-acid peptide that has been widely found to regulate energy homeostasis, glucose and lipid metabolism, and its receptor, GHS-R1a has been expressed in rodent and human adipocytes (Kim et al., 2004; Rodriguez et al., 2009; Miegueu et al., 2011). Our study demonstrated that ghrelin promoted the proliferation of mouse 3T3-L1 and human primary preadipocytes by increasing IGF-1 expression, and IGF-1 also enhanced the proliferation of murine and human cells. In contrast, IGF-1 promoted differentiation, whereas ghrelin inhibited the differentiation and lipid accumulation of adipocytes by downregulating the expression of the transcription factors PPARγ2 and C/EBPα.

Substantial evidence has shown that ghrelin is capable of stimulating 3T3-L1 cell growth (Kim et al., 2004; Liu et al., 2009; Miegueu et al., 2011), and it significantly increased cell numbers at concentrations from 10^–7^ to 10^–15^mol/L (equal to 330 to 3.3 × 10^–6^ ng/ml), which is consistent with our results. The study performed by Liu et al. (2009) showed that after 24 h of treatment, ghrelin (10^–7^, 10^–11^ to 10^–15^mol/L) promoted 3T3-L1 preadipocyte proliferation, and 10^–9^ and 10^–11^mol/L ghrelin also presented a stimulatory effect at 6 h. In our study, we found that the 10 ng/ml ghrelin stimulated 3T3-L1 cell growth at 24 h. A similar effect on human primary preadipocyte proliferation was also observed, but it was at a higher concentration (1000 ng/ml) compared with 3T3-L1 cells. Because the expression of ghrelin receptor-GHSR has been found in both human and rodent adipocytes, an explanation may be the difference in the receptor number and affinity of these two species. Rat ghrelin was used in our present study, and it may have higher affinity with the receptors of murine 3T3-L1 preadipocytes than human preadipocytes (Kos et al., 2009; Rodriguez et al., 2009; Gurriaran-Rodriguez et al., 2011). In addition to the promotional effect on proliferation of mouse 3T3-L1 and human primary preadipocytes, ghrelin is capable of enhancing the growth of other types of cells, such as cardiomyocytes, osteoblasts, murine T cells, and gastric and colorectal cancer cells. In these cells, ghrelin promotes proliferation in a dose-dependent or time-dependent manner (Pettersson et al., 2002). The doses and action times are distinguished by the type of cells; for example, ghrelin significantly enhanced cell growth at concentrations from 1 to 100 nmol/L (equal to 3.3 to 330 ng/ml) in gastric cancer cells (Tian et al., 2013), but 3 to 30 μmol/L (equal to 9.9 to 99 × 10^3^ ng/ml) in cardiomyocytes (Pettersson et al., 2002). Considering the action time of ghrelin, murine T cells with ghrelin generally exhibited greater proliferation than the controls at 12 to 48 h (Lee et al., 2014). These studies suggest a wide growth-promotional effect of ghrelin on cells and tissues.

Previous studies have shown that ghrelin upregulated c-Myc protein, a cell cycle activator inducing transition from the G1 phase to S phase, and this mitogenic effect might be due to the activation of the phosphatidylinositol 3-kinase (PI3K)/Akt and ERK1/2 pathways (Kim et al., 2004; Liu et al., 2009; Miegueu et al., 2011). In our study, we found that ghrelin promoted mouse 3T3-L1 and human primary preadipocyte proliferation by directly upregulating the expression of IGF-1, and it was further confirmed by using IGF-1 antibody, which abolished the stimulatory proliferation action of ghrelin in both murine and human preadipocytes. The results of the qRT-PCR showed that the mRNA levels of cyclin genes cyclin D1, E1, B2 and apoptosis genes BAX, Bcl-2, Bcl-xL had no significant difference in ghrelin and control groups. These findings suggested that the stimulatory effect of ghrelin may be not by increasing cyclins and decreasing apoptosis. The addition of IGF-1 to preadipocytes could also promote cell growth. Studies performed by Kim et al. (2004) also support our results, showing that IGF-1 could increase the number of 3T3-L1 preadipocytes. Ku et al. (2012) found that IGF-1 suppressed apoptosis induced by serum starvation.

Several studies have reported that ghrelin stimulates the differentiation of mouse 3T3-L1 preadipocytes and enhances lipid accumulation by increasing the expression of PPARγ2 and C/EBPα (Kim et al., 2004; Liu et al., 2009; Miegueu et al., 2011). In these studies, the cells were supplemented with ghrelin at concentrations of 10^–11^ to 10^–13^ mol/L (equal to 0.033 to 3.3 × 10^–4^ ng/ml) for 6 or 9 days. The stimulatory effect of ghrelin was also observed in human visceral adipocytes at concentrations of 10 to 1000 pmol/L (equal to 0.033 to 3.3 ng/ml) (Rodriguez et al., 2009). In contrast, we found that ghrelin inhibited the differentiation of 3T3-L1 and human primary preadipocytes at 0.1, 10, and 1000 ng/ml, and it suppressed the expression of lipogenesis enzymes. RT-PCR further suggested that the mechanism may be the downregulation of the adipogenic factors PPARγ2 and C/EBPα. A study performed by Zhang et al. (2004) supported our finding, reporting that 10^–6^mol/L (equal to 3300 ng/ml) inhibited preadipocytes differentiation. The stimulatory effect of ghrelin was observed at concentrations of 10^–11^ to 10^–13^ mol/L in other studies, whereas 0.1 to 1000 ng/ml ghrelin inhibited differentiation in our study. A possible explanation may be that ghrelin is able to promote differentiation at low doses but markedly suppresses adipogenesis at the high doses. Interestingly, we found that ghrelin modulated proliferation by increasing IGF-1 levels (Figure 3), whereas IGF-1 promoted differentiation (Figures 5G,H), and another study also found that IGF-1 induced adipogenesis (Smith et al., 1988). Ghrelin may downregulate IGF-1 expression to inhibit the differentiation of preadipocytes, indicating it may have different effect on IGF-1 in cell proliferation and differentiation.

In the present study, we demonstrated that ghrelin downregulated the expression of lipogenesis enzymes, thereby reducing the lipid content in adipocytes. Additionally, ghrelin has been reported to have other effects on adipocytes, such as inhibition of lipolysis and anti-inflammation. In this regard, ghrelin suppresses lipolysis by activating of PI3Kγ/Akt and phosphodiesterase (PDE) (Baragli et al., 2011; Miegueu et al., 2011) in mouse adipocytes and decreases aquaporin-7, a glycerol channel for lipid efflux, in human adipocytes (Rodriguez et al., 2009). Additionally, ghrelin also plays roles in anti-inflammatory processes by repressing TNF-α-induced apoptosis and autophagy in adipocytes (Rodriguez et al., 2012), so ghrelin may be beneficial for human obesity and obesity-associated type 2 diabetes.

## Conclusion

Ghrelin enhanced the proliferation of 3T3-L1 and human primary preadipocytes by increasing the expression of IGF-1. Additionally, ghrelin inhibited the differentiation of mouse and human preadipocytes by downregulating PPARγ2 and C/EBPα levels. As a result, lipid accumulation and lipogenic enzymes consistently decreased.

## Data Availability Statement

All datasets generated for this study are included in the manuscript/Supplementary Files.

## Author Contributions

HM did the cell experiments, analyzed data, and wrote the primary manuscript. HP supervised the experiments. LW and HY helped to analyze data. HZ designed the experiments and did the cell experiments. FG supervised the whole experiments and revised the primary manuscript. All authors contributed to manuscript revision, read and approved the submitted version.

## Conflict of Interest

The authors declare that the research was conducted in the absence of any commercial or financial relationships that could be construed as a potential conflict of interest.
