# Supplementary material for: Ghrelin Promotes Proliferation and Inhibits Differentiation of 3T3-L1 and Human Primary Preadipocytes
Source: Front Physiol. 2019 Oct 11;10:1296. doi: 10.3389/fphys.2019.01296 (PMC6798085; doi:10.3389/fphys.2019.01296)
Supplement: Supplementary file 1 [file Table_1.docx]

**Table S1** Clinical details of three patients undergoing surgery

|  | Age | Gender | BMI（Kg/m^2^） | Diagnosis |
| --- | --- | --- | --- | --- |
| 1 | 23 | F | 22.1 | appendicitis |
| 2 | 36 | M | 26.5 | cholelithiasis |
| 3 | 58 | F | 31.1 | colon benign neoplasm |
